# Development of Small Diameter Nanofiber Tissue Engineered Arterial Grafts

**DOI:** 10.1371/journal.pone.0120328

**Published:** 2015-04-01

**Authors:** Hirotsugu Kurobe, Mark W. Maxfield, Shuhei Tara, Kevin A. Rocco, Paul S. Bagi, Tai Yi, Brooks Udelsman, Zhen W. Zhuang, Muriel Cleary, Yasuko Iwakiri, Christopher K. Breuer, Toshiharu Shinoka

**Affiliations:** 1 Nationwide Children’s Hospital, Columbus, Ohio, United States of America; 2 Yale University School of Medicine, New Haven, Connecticut, United States of America; North Carolina A&T State University, UNITED STATES

## Abstract

The surgical repair of heart and vascular disease often requires implanting synthetic grafts. While synthetic grafts have been successfully used for medium-to-large sized arteries, applications for small diameter arteries (<6 mm) is limited due to high rates of occlusion by thrombosis. Our objective was to develop a tissue engineered vascular graft (TEVG) for small diameter arteries. TEVGs composed of polylactic acid nanofibers with inner luminal diameter between 0.5 and 0.6 mm were surgically implanted as infra-renal aortic interposition conduits in 25 female C17SCID/bg mice. Twelve mice were given sham operations. Survival of mice with TEVG grafts was 91.6% at 12 months post-implantation (sham group: 83.3%). No instances of graft stenosis or aneurysmal dilatation were observed over 12 months post-implantation, assessed by Doppler ultrasound and microCT. Histologic analysis of explanted TEVG grafts showed presence of CD31-positive endothelial monolayer and F4/80-positive macrophages after 4, 8, and 12 months *in vivo*. Cells positive for α-smooth muscle actin were observed within TEVG, demonstrating presence of smooth muscle cells (SMCs). Neo-extracellular matrix consisting mostly of collagen types I and III were observed at 12 months post-implantation. PCR analysis supports histological observations. TEVG group showed significant increases in expressions of SMC marker, collagen-I and III, matrix metalloproteinases-2 and 9, and itgam (a macrophage marker), when compared to sham group. Overall, patency rates were excellent at 12 months after implantation, as structural integrity of these TEVG. Tissue analysis also demonstrated vessel remodeling by autologous cell.

## Introduction

Synthetic grafts are widely used in cardiovascular surgery as conduits for arterial bypass in large- and medium-sized vessels. Unfortunately, use of synthetic grafts in small diameter applications (<6mm) has been limited [1] due to high rates of early occlusion secondary to thrombosis after implantation [2].

Currently, autologous veins and arteries are used for small diameter arterial grafts. However, use of autologous grafts leads to prolonged operative time, increased risk of peri-operative infection, and an inherent anatomic limitation in the number of grafts that can be harvested from a given patient. Therefore, the need for small diameter synthetic grafts in cardiovascular surgery remains high for acquired heart and peripheral artery disease [3–5].

Tissue engineered vascular grafts (TEVG) offer the potential for constructing the ideal conduit for small diameter arteries. The ideal TEVG should be readily available (“off-the-shelf”), resistant to thrombosis, resistant to infection, capable of neovessel formation without neointimal hyperplasia and stenosis, capable of autologous repair, growth, and remodeling, and resistant to aneurysmal dilatation and ectopic calcification. In addition, TEVG should be capable of surgical handling for implantation and have biomechanical properties consistent with that of native artery. To the latter point, the temporal development of extracellular matrix within the scaffold that is capable of withstanding intraluminal systemic arterial pressures as the synthetic scaffold gradually degrades is an absolute necessity for the clinical translation of TEVG.

Our team has developed a successful TEVG that is used in low pressure systems and has been applied clinically as an extracardiac total cavopulmonary connection in humans as early as 2001 [6]. Clinical results, both short- and long-term, have been encouraging [7, 8].

Herein, we report our study in which we constructed TEVG composed of polylactic acid and spun into tubular nanofiber scaffolds with extremely small diameter lumens (0.5 to 0.6 mm), and successfully implanted these grafts as aortic interposition conduits in mice with long-term survival and with no instances of graft stenosis or aneurysmal dilatation. Further, presence of remodeled TEVG at 1 year after implantation was evident as indicated by cellular infiltration, extracellular matrix deposition, and tissue remodeling by 12 months.

## Methods

### Study Design

The objective of this study was to perform an observational study over 1 year evaluating the efficacy of a novel electrospun nanofiber graft for implantation as an arterial conduit in mice. We implanted grafts in 25 mice, a number that allowed us to sacrifice mice at different timepoints. All implantations were included in this study, with no instances of data exclusion. Outliers were included in the data analysis. This was an unblinded study.

### Animals experiment’s protocol and treatment

All animals received humane care in compliance with the National Institutes of Health (NIH) Guide for the Care and Use of Laboratory Animals. The Institutional Animal Care and Use Committee at Yale University approved the use of animals and all procedures described in this study.

For surgical implantations, the mice were anesthetized with Ketamine (Hospira, Inc., Lake Forest, IL 60045) and Xylazine (Akorn, Inc., Decatur, IL 62522). Ketoprofen (Fort Dodge Animal Health, Fort Dodge, Iowa 50501), an analgesic, was injected before anesthesia. Motrin water was provided for 48 hours after surgery.

We examined the mice daily following surgery for 7 days to follow surgical recovery, and monitored for hindlimb paralysis, wound integrity/bleeding, hygiene, dehydration, and overall well-being. The animals were humanely euthanized with CO_2_ if they met the following clinical criteria: 1) Prolonged (greater than 48 hours) in appetence and/or clinical dehydration, 2) Inability to ambulate, preventing ready access to food or water.

### Scaffolds

**Fig 1:**
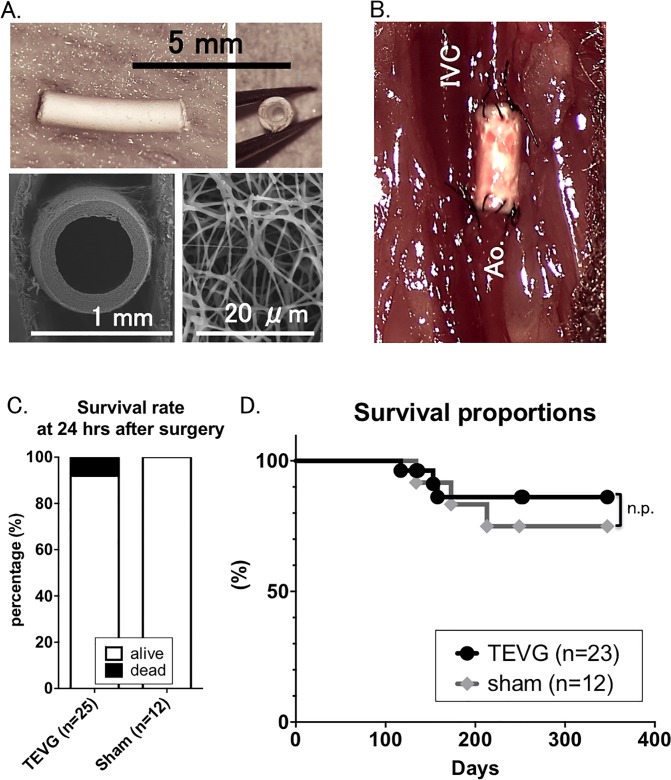
TEVG implantation was successful as indicated by a similar survival rate at 12-month post-operation in TEVG group to sham group. A. Structure of TEVG. Implanted TEVG was composed of biodegradable electrospun polylactic acid (PLA) nanofibers with a length of approximately 3mm and inner luminal diameter 500–600 μm. Scanning electron microscopy demonstrates macro- and micro-arrangement of graft. **B.** TEVG after surgical implantation (IVC: inferior vena cava; Ao: aorta). **C.** Survival rate at 24 hours after surgery. 25 TEVGs were implanted in C17SCID/bg mice. 12 syngeneic mice underwent sham operations. >90% of mice implanted with TEVG survived 24 hours after TEVG implantation. Peri-operative mortality was due to bleeding or thrombosis. **D.** Kaplan-Meier survival curve during the 12 months of experiment. Similar survival rate was seen between two groups over the course of 12 month-experiment.

Scaffolds were composed of polylactic acid (PLA) and spun into porous nanofiber conduits using electrospinning technology (Gunze Corporation, Tokyo, Japan). Briefly, a solution of PLA in HFIP (1,1,1,3,3,3-Hexafluoro-2-propanol) was placed in a syringe with a conductive needle. Elctrospinning voltage of 10–15 kV was applied to the needle. Nano-fibers were fabricated into a tube-like construct by collecting onto a rotating metallic mandrel [9, 10]. Each scaffold was 3 mm in length, 150μm in wall thickness, 78% in porosity and 0.27 g/cm^3^ in density. Inner luminal diameters were between 500 and 600 μm. Each scaffold construction was evaluated using scanning electron microscopy, following sputter coating with palladium (FEI, Hillsboro, OR) (Fig. 1A) [11, 12].

### Graft Implantation

Grafts were implanted using standard microsurgical technique. 8-week-old female C17SCID/bg mice were implanted with infra-renal aortic interposition conduits using 10-0 nylon suture for the end-to-end proximal and distal anastomoses with about 3 mm in length (Fig. 1B). A total of 25 grafts were implanted. Grafts were harvested at months 4 (n = 7), 8 (n = 7) and 12 (n = 9) after implantation for analysis. Post-operatively, no anti-platelet or anti-coagulant agents were used.

Twelve syngeneic mice underwent sham operations, which exposed and isolated the infra-renal aortic aorta followed by closure of the abdomen.

### Ultrasound

Serial ultrasonography (Vevo Visualsonics 770; Visualsonics, Toronto, ON, Canada) was used to monitor grafts after implantation. Prior to ultrasonography, mice were anesthetized with 1.5% inhaled isoflurane. Graft luminal diameter was determined sonographically and patency was determined by measuring flow velocity proximal and distal to the graft.

### Contrast-enhanced MicroCT angiography

Under anesthesia, *in vivo* microCT angiography was performed with the GE eXplore Locus *in vivo* microCT scanner (GE Healthcare, Milwaukee, WI, USA). MicroCT data were acquired with an x-ray source of 70 kVp tube voltage, 32 mA tube current, 4×4 detector binning model, 16 milliseconds exposure per frame, 70 gain, and 20 offset for contrast-enhanced CT acquisitions. One minute prior to acquisition, animals were given an intra-jugular 0.3 cc bolus of Ultravist (370 mgI/ml, Bayer Healthcare, Wayne, NJ). A single frame of 220 projections for 42 seconds of continuous x-ray exposure was used. Volumetric microCT images were reconstructed in a 360 × 185 × 505 format with voxel dimensions of 98.4 ×98.4 × 98.4 μm^3^ using a Feldkamp algorithm with calibrated Hounsfield units (HU).

MicroCT data was transferred to the Advanced Workstation (version 4.4; GE Healthcare) for further reconstruction and quantitative analysis. Sites of anastomosis were approximated. A single operator performed all image analysis. Grafts were identified by the software and manually confirmed. Measurements of graft length, inner luminal diameter, and graft volume were performed. Similar measurements were performed on adjacent aortas in mice implanted with grafts as well as in controls having undergone sham operation.

### Histology

Grafts were harvested at 4, 8 and 12 months were fixed in 4% para-formaldehyde (PFA) and embedded in paraffin. Five-micron thick sections were then stained using standardized techniques for hematoxylin and eosin (H&E), Masson’s Trichrome (collagen), Movat’s, and Elastica van Gieson (EVG) (elastin).

### Immunohistochemistry

Identification of macrophages and matrix metalloproteinase-2 (MMP-2) was done by immunohistochemical staining of the paraffin-imbedded explant sections with rat-anti-mouse F4/80 (1:1000, AbD Serotec, Oxford, UK), rabbit-anti-human matrix metalloproteinase-2 (MMP-2, 1:500, Abcam, MA, USA), rabbit-anti-human CD 31 (1:50, Abcam). Antibody binding for F4/80 and MMP-2 was detected using biotinylated goat-anti-rat IgG (1:200, Vector, Burlingame, CA, USA) and biotinylated goat-anti-rabbit IgG (1:200, Vector), respectively. This was followed by binding of streptavidin-horse radish peroxidase (HRP) and color development with 3,3-diaminobenzidine (DAB).

### RNA Isolation and RT-PCR

TEVG collected at 4, 8, and 12 months after implantation and native aortas were frozen in optimal cutting temperature (OTC) compound (Tissue-Tek; Sakura Finetek, Torrance, CA, USA), and sectioned into twenty 30 μm sections using a Leica CM 1950 cryostat (Leica biosystems, Wetzlar, Germany). Excess OCT compound was removed by centrifugation in PBS. RNA was extracted and purified using the RNeasy mini kit (Qiagen, Venlo, The Netherlands). RT-PCR was performed using predeveloped assay reagents (Applied Biosystems, Carlsbad, CA, USA), as described previously [13]. Primers for the following genes were purchased from Life Technologies (Carlsbad, CA, USA): vimentin (vim; Mm01333430_m1), elastin (eln; Mm00514670_m1), collagen type I (col1a1; Mm00801666_g1), collagen type III (col3a1; Mm01254476_m1), EphrinB2 (Efnb2; Mm01215897_m1), eNOS (Nos3; Mm00435217_m1), Macrophage (Itgam; Mm00434455_m1), MMP-2 (Mmp2; Mm00439498_m1), MMP-9 (Mmp9; Mm00442991_m1). HPRT1 (Hprt; Mm00446968_m1) was used as a housekeeping gene.

### Immuno-fluorescent staining for whole mount TEVG/aorta

Explanted TEVG were cut longitudinally and fixed with stainless steel insect pins on a silicon block. Tissue was then fixed with 4% PFA/phosphate buffered saline (PBS) at 4 degrees Celsius (°C) for 30 minutes, after which tissues were washed in PBS. Tissue was then incubated in a 0.3%-TritonX(TX)100/2% bovine serum albumin (BSA)/PBS solution at room temperature for 15 minutes to achieve permeabilization.

Next, tissues were incubated with primary antibodies, including VE-cadherin (1:100, Santa Cruz Biotechnology, Inc., Dallas, TX, USA), eNOS (1:10, Novus Biologicals, Littleton, CO, USA) overnight at 4°C. The following day, vessels were washed with PBS and incubated with secondary antibodies conjugated to Alexa Fluor 488 or 568 (1:500, Life Technologies) for 3 hours. Finally, vessels were washed with PBS, mounted with media containing DAPI (Invitrogen/Life Technologies), and evaluated using fluorescent microscope (Eclipse E800; Nikon).

### Immuno-fluorescent staining for cross section

Mice were perfused with PBS through left ventricle to flush blood, followed by 4%-PFA/PBS. Aorta was harvested and further fixed in 4% PFA/PBS at 4°C for 2–3 hours. Vessels were then incubated in a 15% sucrose/PBS solution at 4°C overnight, frozen with OCT compound, and cut 8–10 μm in each slice. After drying and removing OCT compound with PBS, 8–10 μm thick sections were incubated with primary antibodies, including von Willebrand factor (vWF) (1:100, DAKO, Carpinteria, CA, USA), CD68 (1:100, AbD), alpha-smooth muscle actin (α-SMA) (1:100, Dako), CD-31 (1:100, BD Biosciences, San Jose, CA, USA), Intracellular Adhesion Molecule-1 (ICAM-1) (1:100, Abcam) and Collagen typeI (1:100, Abcam), with 5% goat serum overnight at 4°C.

The following day, these sections were washed with PBS and incubated with secondary antibodies conjugated to Alexa Fluor 488 or 568 (1:200, Life Technologies) for 1 hour. Finally, vessels were washed with PBS, mounted with media containing DAPI (Invitrogen/Life Technologies), and evaluated using fluorescent microscope (Eclipse E800; Nikon).

### Statistical Analysis

Results are expressed as mean ± standard deviation. The number of experiments is shown in each case. Univariate analysis was performed using student’s *t* test for continuous variables with normal distribution and Chi-square for dichotomous variables. The statistical significance of differences between groups was analyzed using the nonparametric Mann-Whitney test for paired data and one-way ANOVA. A probability value of less than 0.05 was considered significant.

## Results

### Survival rate of TEVG-implanted group was similar to that of sham group

Survival rate at 24 hours after surgery was 92.0% in TEVG-transplanted group, while 100% in sham group (Fig. 1C). Two of 25 implanted mice died in perioperative time, as a result of bleeding from anastomosis and as determined by necropsy. There were no evidence of aneurysmal change or acute occlusion of implanted grafts.

Survival rate at 12-month post-operation was 91.6% in TEVG group and 83.3% in sham group) (no statically significance between both groups, Fig. 1D). Necropsies and tissue analysis were performed on all mice to clarify the cause of death, but there was no evidence of aortic aneurysmal change or stenosis of graft in any mice. Consequently, we can only assume death was of random, natural causes over the course of 12 months, with typical lifespan of less than two years.

### Luminal patency and laminar flow in all TEVG were satisfied results by 1-year follow-up

**Fig 2:**
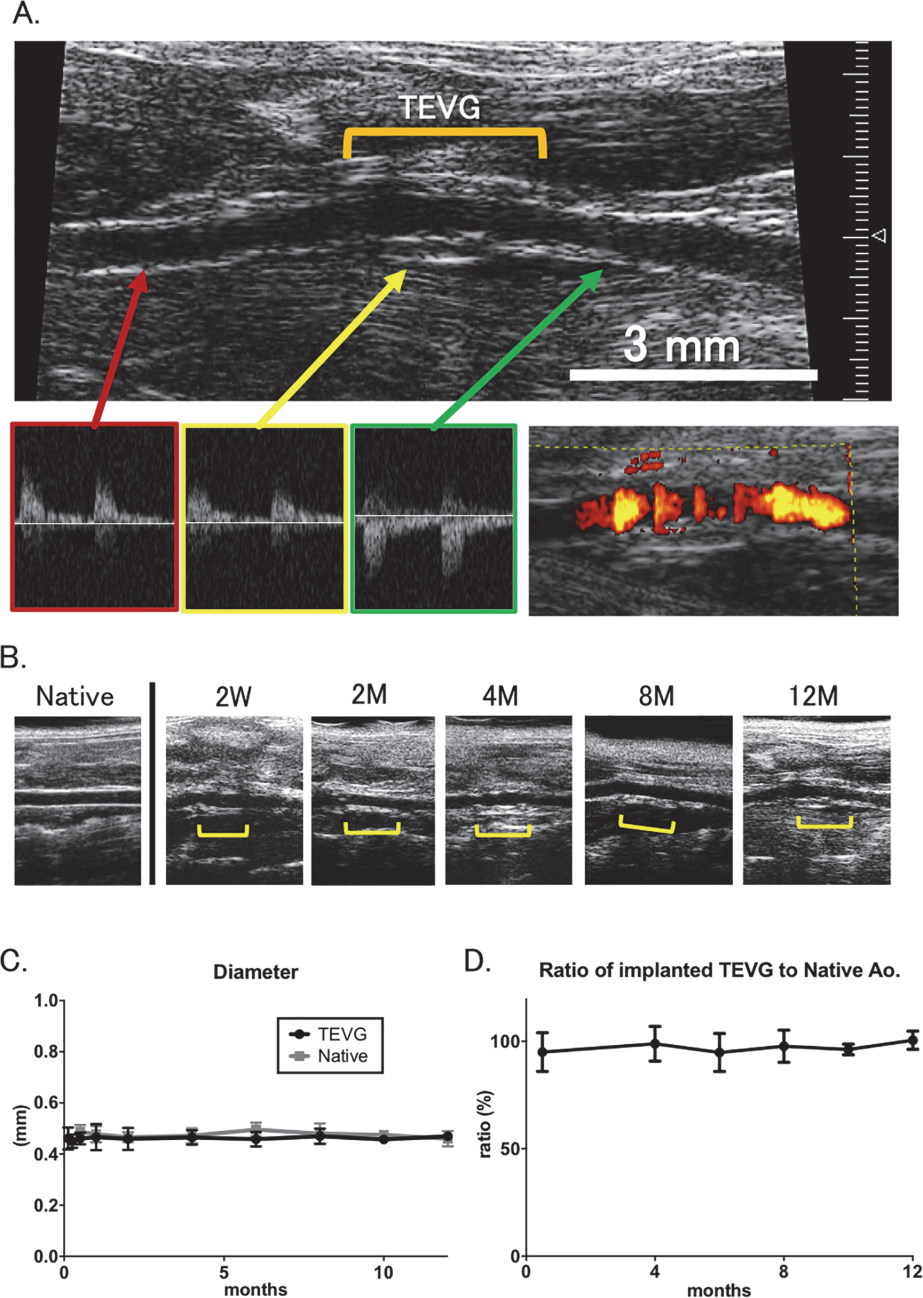
Luminal patency and laminar flow were similar between TEVG and native aorta over the course of the 12 month experiment. **A**. Doppler ultrasound examinations were performed on tissue engineered vascular grafts from 2 weeks to 12 months after implantation. Doppler signals proximal to the graft, distal, and within the graft showed propagation of systemic arterial impulse through the graft. **B.** *In vivo* microCTs were performed at 4, 8, and 12 months. Using image analysis software, luminal volume measurements were recorded and then standardized to a 3mm segment. **C. & D.** When compared to native Aorta in mice having undergone sham operation, there was no significant difference in luminal diameter between both groups and in ratio of TEVG to native aorta at any of the time points.

Implanted TEVGs were serially monitored by ultrasound to assess changes in both graft patency and diameter for up to 12 months (Fig. 2A, B). Average luminal diameter was 0.46 ± 0.04 mm at 3 days (n = 25), 0.45 ± 0.03 mm at 1 weeks (n = 24), 0.46 ± 0.02 mm at 2 weeks (n = 10), 0.47 ± 0.05 mm at 1 months (n = 15), 0.46 ± 0.04 mm at 2 months (n = 8), 0.47 ± 0.03 mm at 4 months (n = 21), 0.46 ± 0.03 at 6 months (n = 12), 0.47 ± 0.03 at 8 months (n = 16), 0.46 ± 0.02 at 10 months (n = 6), and 0.47 ± 0.01 at 12 months (n = 8) (Fig. 2C).

Native aortas were also monitored by ultrasound. Average luminal diameter throughout experimental time course was 0.49 ± 0.03 mm at 2 weeks (n = 5), 0.48 ± 0.03 mm at 1 month (n = 5), 0.47 ± 0.02 mm at 2 months (n = 5), 0.47 ± 0.03 mm at 4 months (n = 5), 0.50 ± 0.03 at 6 months (n = 5), 0.48 ± 0.04 at 8 months (n = 5), 0.48 ± 0.02 at 10 months (n = 5), and 0.46 ± 0.03 at 12 months (n = 5) (Fig. 2C).

Ratio of luminal diameter in implanted TEVG to that in native aorta was also calculated up to 12 months. Average ratio was 95.0 ± 9.0% at 2 weeks, 98.9 ± 8.1% at 4 months, 94.8 ± 8.8% at 6 months, 97.8 ± 7.5% at 8 months, 96.2 ± 2.5 at 10 months, and 100.5 ± 4.3% at 12 months (Fig. 2D).

### Aneurysmal Dilatation or Stenosis was absent in TEVG 12 months after implantation

**Fig 3:**
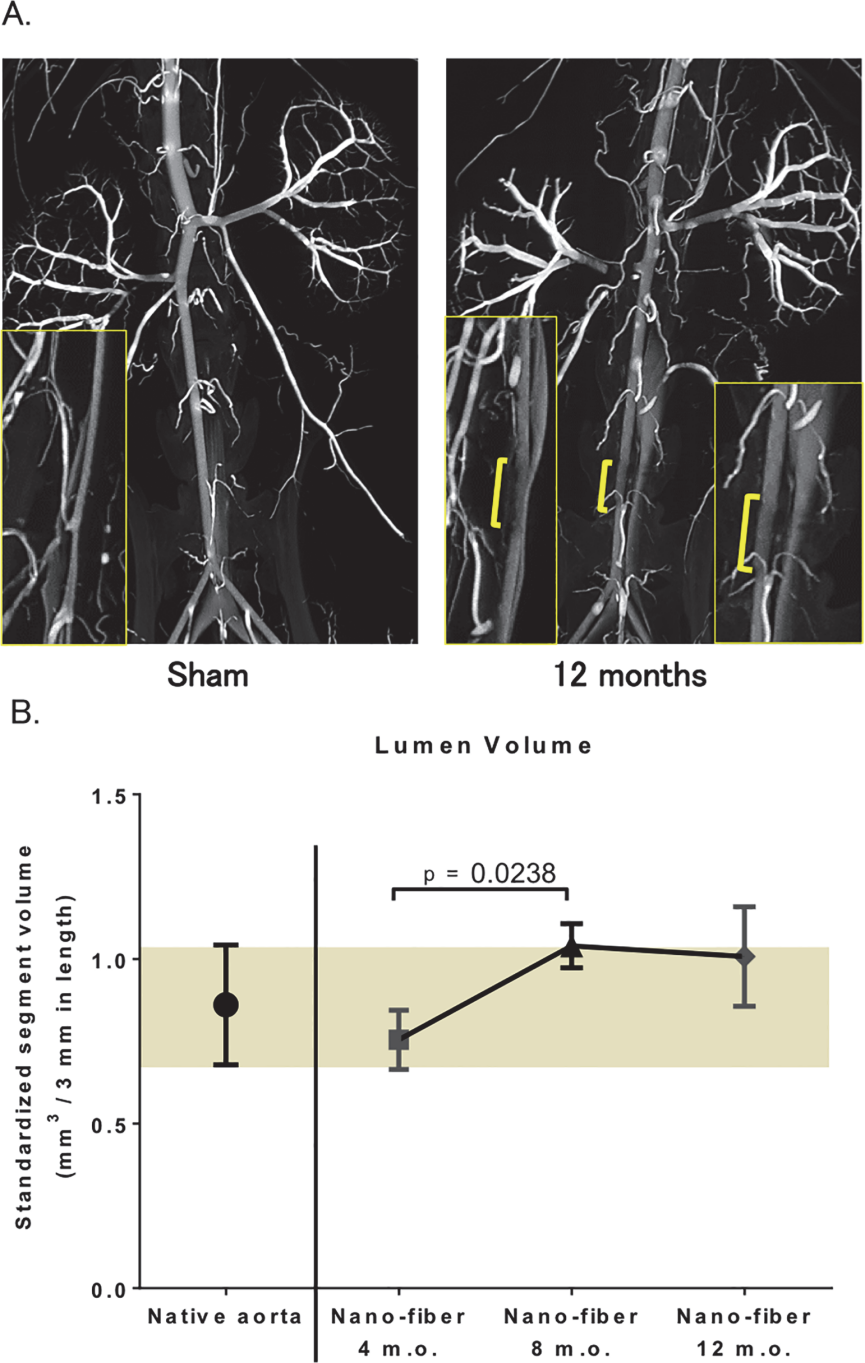
Assessment of TEVG morphometry using microCT angiography demonstrated absence of aneurysmal dilatation or stenosis 12 months after Implantation. **A.** High resolution post-mortem microCT angiography at 12 months post-implantation. The results indicated smooth endoluminal surface and absence of aneurysmal dilatation or stenosis. Yellow bar indicates approximate location of tissue engineered vascular graft. TEVG resemble native aorta on microCT throughout the 12 month-experiment. (n = 5 in each group). **B.** Luminal volume. MicroCT image processing software was used for the calculation. Because of the difficulty in identifying proximal and distal anastomoses, graft luminal volumes were standardized to a 3mm segment. No significant changes were noted over the course of the experiment.

*In vivo* microCT angiography was performed at months 4 (n = 5), 8 (n = 5), and 12 (n = 5). Age-matched controls were also analyzed at each time point. All TEVGs demonstrated luminal patency at each time point without evidence of aneurysmal dilatation or stenosis (Fig. 3A). Due to difficulty in identifying sites of anastomosis, the length of the TEVG was approximated. Luminal volume measurements were performed and analyzed using a standard graft length of 3mm (native Aorta: 0.861 ± 0.182 mm^3^; TEVG: 0.7545 ± 0.090 mm^3^ at 4 months, 1.040 ± 0.067 mm^3^ at 8 months and 1.008 ± 0.151 mm^3^ at 12 months) (Fig. 3B).

### Active vessel remodeling and neo-vessel formation was evident at 12 months after implantation

**Fig 4:**
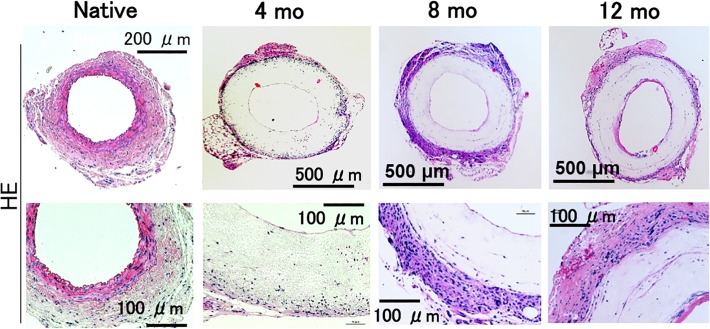
Histological assessment demonstrated active vessel remodeling and neo-vessel formation at 12 months after Implantation. Histologic analysis of TEVG was performed by Hematoxylin and Eosin staining at 5x (top) and 20x (bottom). At 4 months, there is a thin endothelium and some cellular infiltration at the periphery of the TEVG. By 8 months, tissue ingrowth at the periphery has increased. By 12 months, tissue ingrowth has increased at the luminal and peripheral surfaces, with increasing cellularity within the graft. (n = 5 in each group.)

**Fig 5:**
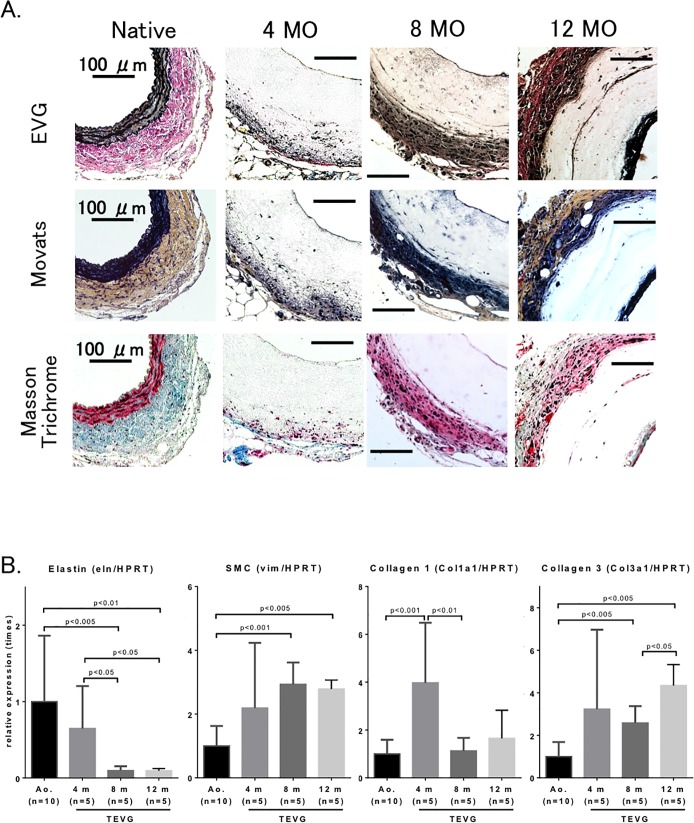
Extracellular matrix deposition was evident in TEVG over the course of the 12-month experiment. **A.** Histologic analysis of graft tissue testing for elastin (EVG: Elastica van Giesen), collagen, elastic and cellular architecture (Movat’s and Masson trichrome) (n = 5 in each group). There is minimal elastin within the graft as compared to that that seen in native aorta. There is presence of endothelial and adventitial cells of the grafts with neo-collagen containing extracellular matrix. **B.** Expression of extracellular matrix components (n = 5–10 in each group). SMC, a marker of smooth muscle cells. Data were normalized using hypoxanthine-guanine phosphoribosyltransferase (HPRT).

Presence of a cellular layer resembling endothelium was seen at our earliest time point, 4 months after implantation. This layer progressively increased in thickness over the course of 12 months, without a concomitant decrease in luminal diameter, indicating cellular ingrowth into the graft rather than neointimal hyperplasia (Figs. 4 & 5A).

Cell infiltration into the graft periphery was robust and increased with time, but lacking in graft interior. By 12 months, a significant amount of scaffold remained. There was a gradual increase in elastin, collagen and connective tissue layer after implantation (Fig. 5A), indicative of an increase in arterial cell number or an increase in arterial cell proliferation.

Qualitative histologic assessment of components of extracellular matrix including vimentin, collagen and elastin showed a gradual increase in collagen deposition within graft tissue. Subjective assessment of increased collagen staining with time was quantitatively confirmed with increased expression of collagen types I and III in grafts. Collagen type I predominated at 4 months, whereas collagen type III was more abundant at 8 and 12 months. There was minimal evidence of elastin formation in TEVG at any time points, nor was there increased gene expression levels (Fig. 5B).

Additionally, we also found micro-calcification changes in two implanted TEVG as determined by von Kossa staining (one at 8 months after implantation, and the other at 12 months), though there were no calcific observations at 4 months. (S1 Fig.)

### Macrophage infiltration and active remodeling of TEVG was most active at 4 months after implantation

**Fig 6:**
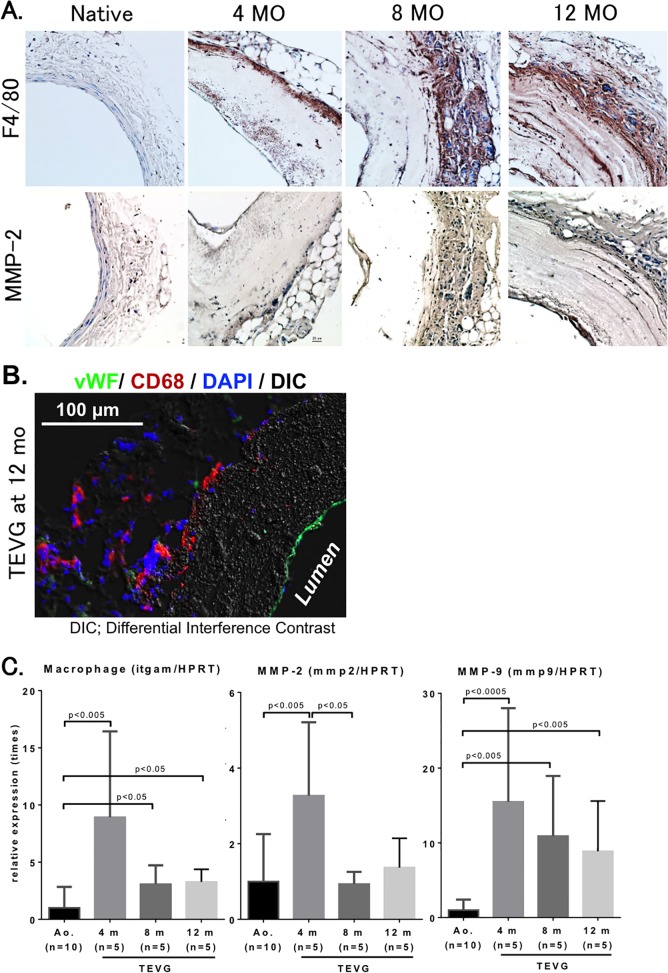
Macrophage infiltration and matrix metalloproteinase activity peaked at 4 months after implantation of TEVG. **A.** Immunohistochemical analysis of macrophage and matrix metalloproteinase-2 (MMP-2) levels. Macrophage infiltration in TEVG was assessed by F4/80 staining, while MMP-2 staining was used for an indicator of TEVG remodeling. (n = 5 in each group). **B.** Endothelial layer staining delineates presence of an endothelium by von Willebrand factor (vWF, green) in implanted TEVG at 12 months after implantation. There is macrophage infiltration of the periphery of the graft (CD68, red) with nuclear (DAPI staining, blue). **C.** Real-time PCR analysis. Data indicate that macrophage infiltration and MMP-2 and -9 expression were most robust at 4 months after implantation (n = 5–10 in each group).

Macrophage infiltration assessed by F4/80 staining within TEVG peaked at 4 months after implantation, with a subjective decrease in time over the course of the 12 month experiment, findings consistent with previous experiments demonstrating that macrophage infiltration is an early and requisite step in transitioning TEVGs from scaffolds to neovessels [14] (Fig. 6A). Macrophage accumulation at the outer area of the TEVG was also demonstrated at 12 months after implantation (Fig. 6B). Presence of CD68 positive cells within the scaffolds suggests the infiltration of macrophages from the outer layer of the scaffolds to within the scaffold, and suggests their role in TEVG remodeling.

Matrix metalloproteinase-2 (MMP-2), an enzyme previously demonstrated to have increased levels of activity early after implantation in murine inferior vena cava TEVG, was present in the scaffold wall at 4 month after implantation [15]. Subjective assessment revealed decreased levels of MMP-2 with time over the course of the 12-month experiment (Fig. 6C).

### An intact and functional endothelium of TEVG mimics that of native aorta

**Fig 7:**
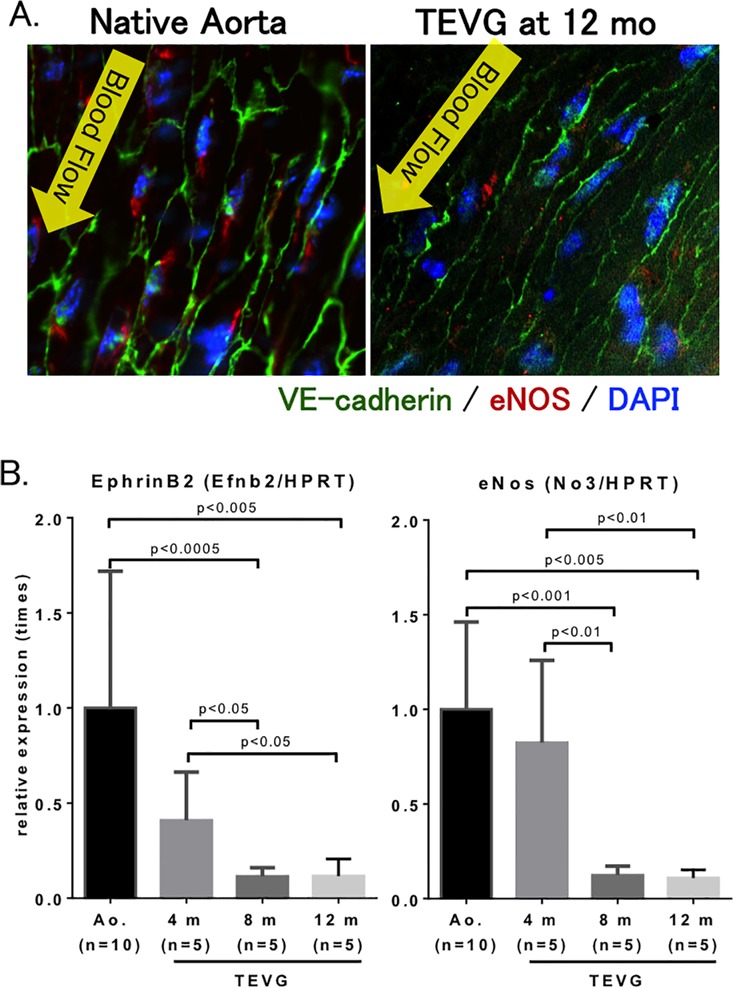
Expression of endothelial markers indicate functional endothelium in TEVG. **A.** Whole mount staining of native aorta and TEVG. VE-cadherin is a marker of cellular borders of endothelial cells (green). Endothelial nitric oxide synthase (eNOS) is a marker of a functional endothelium (red). DAPI is a nuclear stain (blue). **B.** Real-time PCR analysis of ephrinB2 and eNOS. Ephrin-B2, a marker of arterial vessels. (n = 5–10 in each group)

Whole mount staining of aortic graft showed presence of VE-cadherin, a marker of the cellular boundaries that line endothelial cells, and endothelial nitric oxide synthase (eNOS), demonstrating presence of a functional endothelium in TEVG at 12 months after implantation (Fig. 7A).

Expression of eNOS was most robust at 4 months after implantation, whereas levels decreased by 8 months. Levels of Ephrin-B2, a marker of arterial identity, were elevated in the TEVG at 4 months, but less than that seen in native aorta. (Fig. 7B)

**Fig 8:**
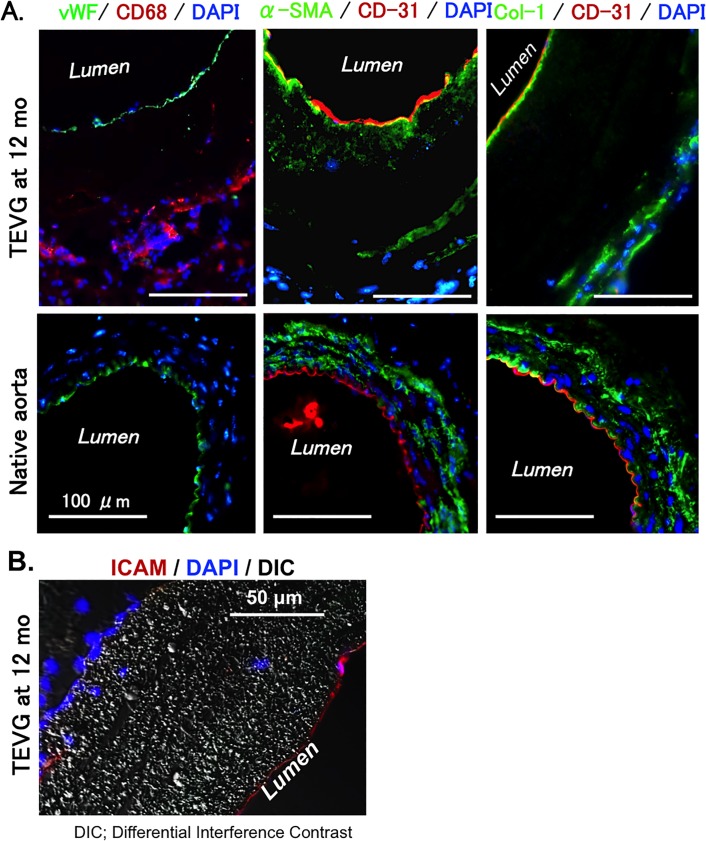
Expression of vascular cell markers in TEVG was similar to that of native aorta. A. Immuno-labeling of vascular cell markers on cross-sections of TEVG and native aorta. Left panel: von Willebrand factor (vWF, green), an endothelial cell marker and CD68 (red), a macrophage marker. Middle panel: CD31 (red), an endothelial cell marker; alpha-smooth muscle actin (α-SMA, green), a marker of smooth muscle cell and myofibroblasts. Right panel: CD31 (red) and collagen type I (Col-1, green). DAPI is for nucleus staining (blue). **B.** Immune-labeling of intracellular adhesion molecule (ICAM) as a marker of endothelial cells.

Further evidence of neovessel formation is demonstrated by positive staining of vWF (endothelium), CD68 (macrophage), alpha-SMA (smooth muscle cells), CD31 (endothelium), and Col-1 (collagen) in the cross-section of the grafts (Fig. 8A). Presence of an endothelial monolayer was demonstrated on the luminal side of the TEVG at 12 months after implantation (Fig. 6B). Alpha-SMA and collagen-1 staining showed that tissue remodeling after implanting occurred from both luminal and outer areas (Fig. 8A), and negative control also showed data to clarify no fluorescence without anti-alpha SMA and DAPI in harvested tissues after implanting (S2 Fig.). Monolayer positive with intercellular adhesion molecule (ICAM) was also observed (Fig. 8B).

## Discussion

In our present study, we constructed TEVG composed of a polylactic acid (PLA) nanofiber generated by electrospinning technology and implanted them as infrarenal interposition aortic conduits in a murine model. Electrospinning, which enables the production of nano fiber-based scaffolds, has emerged as a promising technique for fabricating arterial scaffolds because of its ability to improve endothelialization [16–18]. We used PLA fibers for graft construction because PLA has a prolonged degradation profile, which allows for increased time for the transition of the burden of graft structural and mechanical integrity to be transitioned from the biodegradable scaffold to the neo-extracellular matrix.

Our results suggest that these TEVG showed vessel remodeling by autologous cells similar to normal vessels over 12 months. Further, peri-operative mortality was minimal and there was no difference in survival between mice implanted with a graft versus mice having undergone sham operation. This excellent survival data are likely related to the complete absence of aneurysmal dilatation or graft stenosis in any of the 25 grafts implanted.

In the context of clinical application of arterial TEVG, the most catastrophic risks of graft implantation are aneurysmal dilatation or graft rupture. For coronary artery bypass grafting in humans, we frequently use autologous vein or arterial grafts with 3–5 mm diameter for aorta-coronary artery anastomosis. Importantly, the larger the vessel radius, the greater the wall tension required to withstand a given internal fluid pressure by LaPlace's Law. It is thus important to continue to investigate the temporal changes in mechanical properties of arterial grafts in order to further optimize arterial TEVG use in clinical applications.

We evaluated our arterial TEVG by implanting into extremely small mouse aortas (0.5–0.6 mm) whereas those used in the study by Wu and colleagues [19] implanted in rat aorta with a diameter of 0.7–0.8 mm. In our study, TEVG were serially explanted from 4 to 12 months after implantation, at which time explanted neovessel showed evidence of cellular infiltration, extracellular matrix composition, and an endothelial layer. We also observed a gradual increased elastic layer on the outer surface of TEVG by 12 months, though gene expression of elastin in implanted TEVG was decreased compared to that in native aorta (Fig. 5). Otherwise, our tissue analyses also showed implanted graft were re-modeled with smooth muscle layer and extracellular matrix (ECM), and gene expressions of SMC, collagen 1 and 3 also supported to be activating in these amplification (Fig. 5). Recruitment of macrophages of the graft was observed as early as 4 months after implantation in this study. Macrophages accumulation in the outer layer (i.e., adventitia) of the graft was increased in a time-dependent manner, though RT-PCR data showed that gene expressions of itgam, MMP-2 and MMP-9 peaked at 4 months after implantation. This dissociation may mean gene expression peaked before accumulation of macrophage and inflammatory responses by histological evaluation. Importantly, by 12 months after implantation, those macrophages move into the scaffold from the outer layer of the graft. This movement of macrophages could be a prerequisite of neo-vascularization of the graft. Macrophages can generate MMP-9 and play important roles in matrix remodeling of vessels [20]. Thus, our data also indicates that accumulation of macrophages may be one of the most important factors in tissue remodeling in implanted TEVG. In this discrepancy of results between tissue analysis and gene expressions of elastin, macrophage, MMP-2 and MMP9, we need more experiments to clarify.

In addition, elastin has been shown to play a critical role in artery structure, and there are some reports that highlight the importance of elastase, cathepsin-S and –K in elastin remodeling [21, 22]. Thus, these proteins are strongly related to cardiovascular development. Additional experiments are needed to clarify the functions of these elastin-related proteins under remodeling after implantation.

The endothelial layer on the inner luminal surface showed a monolayer of CD31 positive cells without a concomitant decrease in luminal diameter, indicating cellular ingrowth into the graft rather than neointimal hyperplasia at 1 year after implantation. This endothelial monolayer in our TEVG was structurally and functionally similar to those endothelial cells in the native aorta; immuno-fluorescent staining showed endothelial cells in TEVG at 12 months after implantation are structurally arranged longitudinally in the direction of flow (Fig. 7), the same as those in native aorta [23, 24]. eNOS expression as a marker of functional endothelial cells was observed in peri-nuclei area, presumably on the Golgi apparatus, which in endothelial cells is typical of eNOS cellular localization [25, 26], however, eNOS expression in TEVG was decreased compared to those in native aorta. This result might show the function of endothelial cells was weakened by its cell differentiation for remodeling [27, 28]. Our results also showed a thin layer of Collagen type I underneath the endothelial layer. The existence of this collagen layer may be indicative of the regeneration of the basement membrane, whose function is to provide anchoring support for the endothelium [24, 29, 30] (Fig. 8). Due to limited size of tissue we were unable to complete staining for collagen IV, but future experimentation will plan for this to better understand regeneration of basement membrane [31].

The source of endothelial cells on the luminal side of the graft remains to be solved. We speculate that endothelial cells on the native vessel adjacent to the sutured graft may migrate and proliferate on the graft, since those endothelial cells on the graft closer to the native vessels have higher eNOS similar to the native vessels and gradually decreases eNOS toward the center of the graft. Previous work in venous grafts supports this hypothesis [32]. Nevertheless, we cannot rule out the possibility that circulating endothelial progenitor cells may contribute to the engraftment of endothelial cells.

Though PLA offers well-established material safety [33] and toughness [34] profile for withstanding systemic intra-luminal arterial pressures, we did observe micro-calcification in TEVG by 12 months in 2 sacrificed mice with implanted TEVG. Development of micro-calcification may be related to the duration after graft implantation, the degradation kinetics of graft material, or the graft pore size. Elucidating the mechanism of graft micro-calcification and identifying methods to prevent it will be necessary in order to continue progress towards clinical application of these arterial TEVG.

Tissue analysis also revealed that PLA nano-fiber scaffold remained up to 1-year after implantation, preventing characterization of TEVG through polymer degradation in a rodent model. This problem has been reported in other evaluations of electropsun scaffolds, and more consideration should be given to the timing and tailoring of polymer degradation [20]. Additionally, it is possible the polymer degradation was delayed in this instance due to the high density of the polymer fabric and limited graft-pore size. Pore-size of our nano-graft was just 3–4 μm, and porosity was 70%. Previously, we also investigated another PLA graft having large pore-size and cellular infiltration was better when compared with a nano-fiber graft [35]. However, in that study the survival rates following implantation were unsatisfactory due to rupture or bleeding. Taken together, future TEVG design should be rationally improved to optimize these parameters.

Herein, we successfully implanted small diameter (0.5–0.6mm) synthetic biodegradable PLA nanofiber tissue engineered vascular grafts as aortic interposition conduits in mice. Long-term survival was excellent with no instances of graft stenosis or aneurysmal dilatation. Cellular infiltration, extracellular matrix deposition, and tissue remodeling revealed evidence of neovessels by 12 months. Further investigation is needed to optimize TEVG pore-size, biodegradable material, degradation time and animal models to improve arterial TEVG towards clinical translation. Additionally, this study made use of a small animal model to evaluate graft function, with luminal diameter of only 0.5mm. While this diameter is too small to imply human use, it enables a worst-case, high throughput testing model for graft materials. Further investigation in large animal model with longer lifespans is needed to fully evaluate temporal degradation characteristics and patency of long term degrading TEVG. We will schedule to do more investigation on this front in near future.

## Limitation

This study featured a use mouse model for vascular graft implantation, however, the typical lifespan of mice is only up to 1.5 years. Thus it is difficult to do long-term experiments in mice with this scaffold. In future, more investigation using animals of greater life span is needed.

We utilized SCID/Bg mice according to our previous experience demonstrating lower rates of TEVG thrombosis and stenosis when implanted in this strain, and this specific model might have affected the results of the current study. Based on this limitation, we have since developed an aortic implantation model for TEVG in the wild type C57BL/6 mouse by using anti-platelet and anti-coagulant drugs to reduce acute thrombosis and stenosis following TEVG implantation.

## Supporting Information

S1 Fig Calcified assessment demonstrated active vessel remodeling and neo-vessel formation at 12 months after Implantation. Von Kossa staining performed for histologic analysis of TEVG of calcification after implantation. Arrows in figure shows micro calcification area.
(TIF)

S2 Fig Negative control for immuno-staining in implanted TEVG. Immuno-labeling of vascular cell markers on cross-sections of TEVG. Anti-CD31 (red) antibody was just used for clarification of endothelial layer.
(TIF)
