# Supplementary figures and images for: Development of Small Diameter Nanofiber Tissue Engineered Arterial Grafts
Source: PLoS One. 2015 Apr 1;10(4):e0120328. doi: 10.1371/journal.pone.0120328 (PMC4382213; doi:10.1371/journal.pone.0120328)

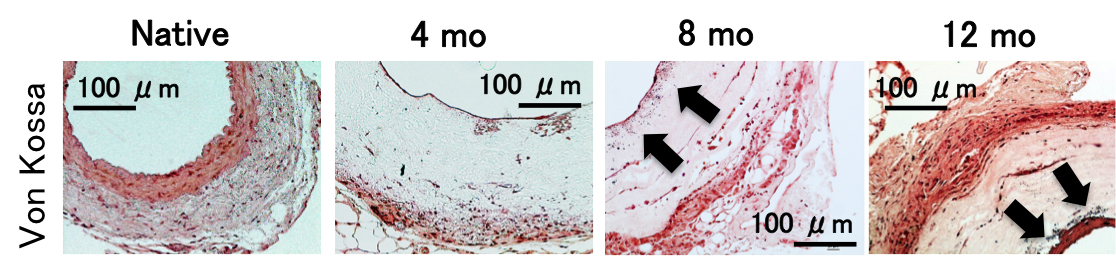

Supplement: S1 Fig — Von Kossa staining performed for histologic analysis of TEVG of calcification after implantation. Arrows in figure shows micro calcification area. (TIF) [file pone.0120328.s001.tif]

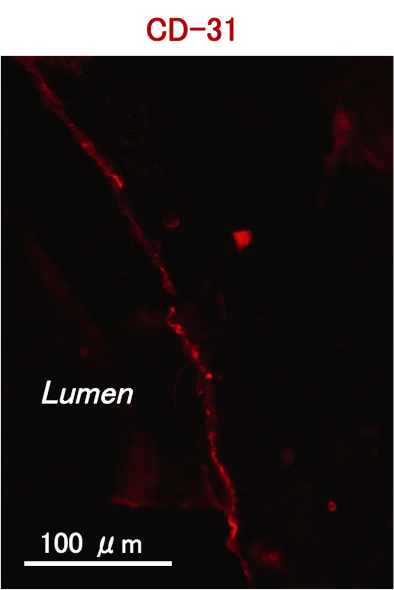

Supplement: S2 Fig — Immuno-labeling of vascular cell markers on cross-sections of TEVG. Anti-CD31 (red) antibody was just used for clarification of endothelial layer. (TIF) [file pone.0120328.s002.tif]
